# Remote sEnsing obServations of source Parameters and data IntegRation at EtnA: the RESPIRA database

**DOI:** 10.1038/s41597-026-07524-5

**Published:** 2026-06-02

**Authors:** Luigi Mereu, Simona Scollo

**Affiliations:** Istituto Nazionale di Geofisica e Vulcanologia, Osservatorio Etneo, Piazza Roma, 2, 95125 Catania, CT Italy

## Abstract

Eruption Source Parameters (ESPs) are fundamental for quantifying paroxysm episodes and are essential inputs for volcanic ash dispersal models used to forecast tephra fallout and assess associated hazard. This is particularly crucial for densely populated regions like Mount Etna, Italy. Since 1986, approximately 150 Etna paroxysm episodes have been monitored using different sensors to estimate these ESPs. While a variety of data is reported in the scientific literature, an integrated dataset has been lacking. In this work, we present an online dataset named RESPIRA: Remote sEnsing obServations of source Parameters and data IntegRation at EtnA. The RESPIRA dataset includes column heights, mass eruption rate and the mode or mean value of total grain-size distribution derived from past publications, from new analyses using different sensors and/or derived from well-known formulation. This comprehensive dataset represents a valuable resource for studying and constraining the paroxysm episodes of Etna, as well as to improve operational services aimed at reducing the hazard.

## Background & Summary

**Fig. 1:**
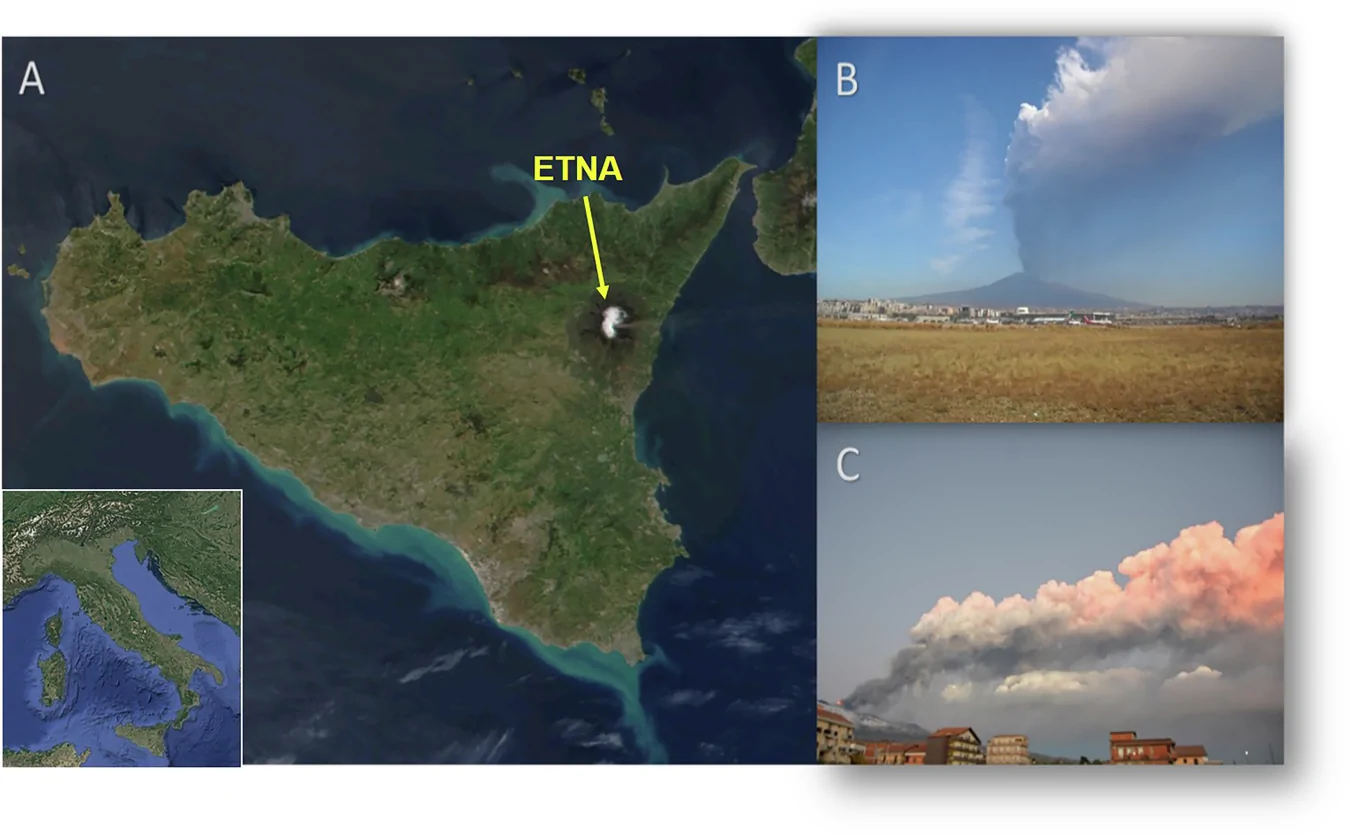
(**A**) Satellite image of Sicily acquired on April 7, 2002 by the MODerate-resolution Imaging Spectroradiometer (MODIS), aboard NASA’s Terra satellite (http://visibleearth.nasa.gov/view_rec.php?id=2631), including an image of Italy taken by Google Maps. (2025), updated 19/03/2026 by https://www.google.com/maps/@42.4602233,13.5477512,6.25z. Eruption columns formed during the paroxysms of B. 8 September 2011 and C. 16 February 2021. Photos by S. Scollo.

Etna, situated on the east coast of Sicily (Italy), is one of the world’s most active volcanoes. Its four main summits - the North-East Crater (NEC), Voragine (VOR), Bocca Nuova (BN), and South-East Crater (SEC) - are situated at an elevation of over 3300 m a.s.l. (Fig. 1A). Historically, the New South-East Crater (NSEC) formed as a pit-crater on the eastern flank of the SEC in 2006, becoming the primary site of paroxysmal activity since 2011^1^. However, since 1 December 2020, the NSEC and SEC are referred to as the South-East Crater (SEC). This change reflects the progressive coalescence of the NSEC with the original SEC, which has eliminated their distinct morphologies^2^. Consequently, this paper makes no distinction between the two cones. Summit craters frequently produce explosive activity, ranging from low-moderate intensity events (e.g. ash emission, strombolian activity) to powerful lava fountains, which are commonly referred to as paroxysm episodes (Fig. 1B,C). At Etna, a ‘paroxysmal episode’ is defined as a short-lived eruptive period characterized by higher intensities and magnitudes than the typical lava effusion and Strombolian activity. These episodes consist of three distinct phases—resumption, paroxysmal, and conclusive—each featuring unique eruptive styles and geophysical signals^3^. The paroxysmal phase is the most intense, involving sustained lava fountains that generate volcanic plumes. Between 1986 and 2025, Etna produced approximately 300 of these events. During the most intense phase, which typically lasts from one to several hours, the eruption columns can rise up to 15 km a.s.l.^4^ generating strong plumes^5,6^. However, in most cases, these paroxysm episodes produced volcanic plumes with heights around 5–8 km a.s.l., resulting in weak volcanic plumes^4,7^. According to^6^, plumes are defined as strong or weak when the characteristic plume velocity is much greater or lower than the ambient wind speed. This definition is widely adopted also to classify volcanic plumes at Etna^4,7^. Tephra ejected during these events poses a severe impact on the population living on the Etna’s slopes, affecting ground transportation and infrastructure^8^. Furthermore, the dispersal of volcanic ash into the atmosphere constitutes a significant hazard to air traffic^4^, sometimes reaching different countries such as Malta^9^ and Greece^10^. For these reasons, continuous monitoring of paroxysm episodes is becoming increasingly important due to the high frequency and intensity of those events.

Eruption source parameters (ESPs) are key parameters for simulating volcanic ash dispersal and fallout. They are crucial for volcanic ash forecasts and are necessary to quantify the hazard associated with tephra dispersal and fallout. Two different types of ESPs have been recognized in literature: 1) those that can be directly measured and 2) those that can be constrained using numerical relationships or through studies of the tephra deposit^11^. In the first category is the column height (CH in km), which can be measured by various sensors, such as radar, lidar, calibrated visible camera and satellite radiometer. The second case concerns the total grain-size distribution (TGSD), that is usually estimated by the analysis of grain-size distribution of samples collected in tephra deposit^7,12^. Deposit-derived ESPs also include estimates of CH, mean MER, and TM. The mass eruption rate (MER), in contrast, can be estimated using retrieval techniques using sensors like camera^13^ and radar^8,14^ or estimated from the CH^15,16^. As ESPs are extensively used for various purposes (e.g. volcanic ash forecasts, estimation of Volcanic Explosive Index (VEI), identification of eruptive style), specific datasets compiling these parameters for volcanoes worldwide have been produced in literature^15–17^. Over the last 30 years, remote sensing systems have greatly enhanced the monitoring and detection capabilities of the eruptive columns generated by paroxysm episodes. A review of the main sensors used to monitor volcanic plumes, spanning from satellite to ground-based sensors, can be found in^18^. This review includes 29 studies on Etna from scientific literature, which is considered one of the best monitored volcanoes in the world. Indeed, various sensor observations have been documented^8,13,19,20^, as well as in reports published by volcano observatories and in the weekly multidisciplinary bulletins and in the Volcano Observatory Notices for Aviation (VONA) issued by the Istituto Nazionale di Geofisica e Vulcanologia, Osservatorio Etneo (INGV-OE)^21^ (see www.ct.ingv.it for reports, bulletins and VONA).

Table 1 reports the sensors used in this paper, along with a brief description of the main characteristics. In this work, we compile a comprehensive dataset of ESPs for the paroxysmal phases of 141 paroxysmal episodes that occurred between 1986 and 2025 at Etna and report them in the RESPIRA repository. The 141 episodes are selected based on the availability of either direct sensor measurements and/or reliable quantitative descriptions found in the scientific literature. The remaining 147 episodes, considering the work of^22^, were not included in the RESPIRA database due to the lack of direct measurements or estimates of at least some ESPs. The collection of the main ESPs for paroxysmal phases significantly contributes to a better understanding and characterisation of the different processes occurring during paroxysmal episodes and the overall explosive eruption behaviour at Etna. This dataset will facilitate statistical analyses of ESP recurrence, the identification of new eruptive scenarios, and the investigation of complex interactions between eruption columns and the atmosphere.

**Table 1:** A brief description of the main characteristics of the different sensors used is reported below.

Sensors	Brief description	Time resolution	Spatial Resolution
Visible calibrated camera^A^	Calibrated cameras named ECVH and EBVH installed on the slopes of Etna are part of the INGV-OE video-monitoring network system.	5 minutes	+/− 500 m
TIR camera^B^	Thermal Infrared Camera (TIR): FLIR A40M	2 frames per seconds	Field of View (FOV): 18°(vertical) 24°(horizontal)
VIS camera^B^	Visible camera (VIS): Vivotec IP8172	1 frame per minutes	Field of View (FOV): 24°-68°(vertical) 33°-93°(horizontal)
Radar DPC^C^	Weather radars operating to C- and X-band managed by the Italian Department of Civil Protection (DPC)	Scanning time of 10 minutes	100-200 m
Radar VDR^D^	Radar system named VolDoRad (VDR), fixed pointing radar operating to L-band	0.2 second	150 m
Satellite radiometer^E^	Spinning Enhanced Visible and Infrared Imager (SEVIRI) is a multichannel scanning radiometer on board of geostationary platform (GEO), that provides image data in Visible and Near-InfraRed (VNIR) and InfraRed (IR) channels	repeat cycle of 15 min and 5 min for High Resolution Visible (HRV) channel	(spatial resolution) is 3 km at nadir and up to 1 km for the High Resolution Visible (HRV) channel

A^7,47^; B^13^; C^57^; D^58^; E^24^.

## Methods

**Fig. 2:**
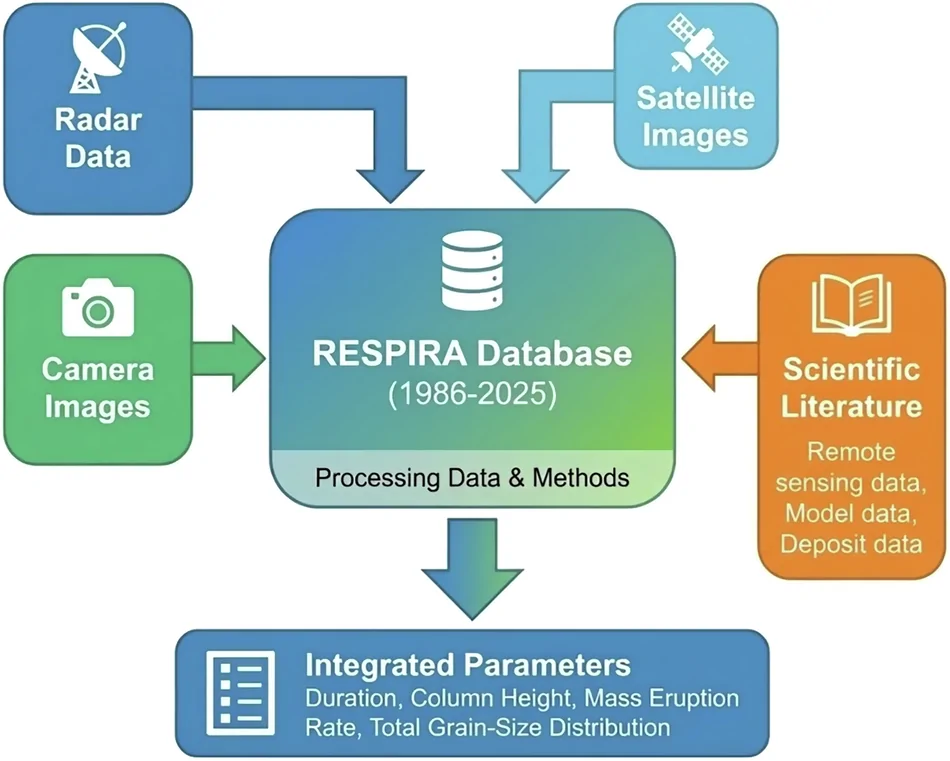
Schematic representation of the workflow used in the RESPIRA database. Literature sources, data processing and applied methods are grouped within the purple box. We identify the radar data, cameras and satellite images in different colored boxes, while scientific literature including modelling, analysis of tephra deposit and remote sensing data are grouped in the orange box. Image improved using Gemini in Google Slides.

This section shortly summarise the methodologies applied to analyse ESPs: the onset (T0) in Coordinate Universal Time (UTC), duration (Dt in minutes), maximum value of CH (in km), maximum value of MER (kg/s), TM (kg) and the mode or mean values of TGSD (φ scale, where φ = -log_2_(D)) is computed as the logarithm of particle diameter D (mm)^23^. Figure 2 presents a schematic diagram showing the workflow and the sequential steps followed in the construction of the RESPIRA database. The final dataset was also compiled from literature sources detailed in References sheet of RESPIRA database^3,16,20,22–45^.

### Duration

Paroxysmal episodes at Etna are typically characterised by three different phases^3^. The first is a ‘resumption phase’, which is marked by a gradual increase in the volcanic tremor amplitude, the onset of the Strombolian activity often including lava flows. This is followed by the ‘paroxysmal phase’ forming a sustained lava fountain. During this phase, there is an increase of lava flow effusion and the formation of eruption columns. Finally, the ‘conclusive phase’ corresponds to a decrease in the explosive activity and the end of the eruption. In this dataset, we consider the duration of the paroxysmal phase for each episode as also reported in^19,22,42^. T0, defined as the onset of the paroxysmal phase, and the duration are taken in our study from scientific literature that include data derived by radar observations and taken by multidisciplinary reports: https://www.ct.ingv.it/index.php/monitoraggio-e-sorveglianza/prodotti-del-monitoraggio/bollettini-settimanali-multidisciplinari) and VONA^7,24^ (https://www.ct.ingv.it/index.php/monitoraggio-e-sorveglianza/prodotti-del-monitoraggio/comunicati-vona).

However, no clear threshold behaviour defines the onset of the paroxysmal phase. Instead, the transition duration scales with eruptive intensity, spanning from 10–15 minutes for high-intensity events to 30 minutes for weaker ones^46^. In contrast, the uncertainty for the conclusive phase is estimated at less than 15 minutes, marked by the absence of a sustained eruption column.

### Column height

The maximum column height at Etna is calculated using three primary complementary methods:

- **Visible camera images**: this method determines the plume height by geometrically intercepting the highest altitude level of the eruption column above the Etna craters. This procedure gives known altitude levels, considering the distance between the camera location and Etna summit craters and the mean of the wind direction within the eruption column^7,47,48^.
- **Thermal Infrared Data using satellite sensors**: this method uses SEVIRI satellite images by identifying the coldest pixel within the volcanic plume near Etna’s summit. In this case, we consider only the images at the wavelength of 10.8 μm, where the imaginary part of the refractive index of silicate (main component of volcanic ash) shows its absorption peak^24,49^. The temperature value (K) of this coldest pixel is then detected, and the corresponding altitude is obtained by matching this temperature with the temperature profile derived from the ERA5 reanalysis data^50^.
- **Radar data**: this involves applying a threshold algorithm to the radar reflectivity measurements taken over the Etna’summit^20,51^. When the measured reflectivity exceeds a specific threshold, it indicates the presence of the dense tephra column, allowing its vertical extent to be estimated.

### Mass eruption rate and total mass

The max MER was retrieved by radar DPC applying the mass continuity equation to tephra concentration values^14,52,53^. This approach allows us to evaluate the time-space variation of the tephra mass within each radar bin volume of the probed volcanic cloud. The max MER was also computed by VDR radar evaluating the mass variation in function of the number of the recorded samples at specific acquisition time intervals and only for the probed range volume above the crater^14^. When the radar observations were unavailable, the MER is derived from the TIR camera, applying the surface flow relation^52,53^. Indeed, the height of the jet region detected by the TIR camera can be converted into the vertical outflow velocity of a pyroclastic material flowing from the crater^54,55^. Moreover, max MER is obtained from the maximum value of CH a.s.l. minus the altitude of the summit crater (H_vent_), using the empirical relation established by^43^: ***MER = ((CH-H***_***vent***_***))/0.159)***^***3.534***^. The Total Mass (TM) is also estimated by radar integrating the MER over the duration of the paroxysmal phase^51,52^. However, these values are generally overestimated and should be treated as upper limits. When available, TM is obtained by the scientific literature.

### Total grain-size distribution

The mode or mean values of TGSD was obtained either directly from scientific literature using the analysis of tephra deposits or through modelling. For the latter, we utilized the model proposed by^44^, which links CH and magma viscosity to the TGSD. Specifically, the mode of the TGSD was estimated using the supplementary spreadsheet (ESM3) provided in Appendix B of^44^. However, based on our dataset’s CH (ranging from 2 to 12 km a.v.v.) and considering the range of the values of Etna magma viscosity (1.9, 2.3, and 2.7 Pa/s), the mode of TGSD is always equal to −1 φ.

## Data Records

The RESPIRA repository is available at Istituto Nazionale di Geofisica e Vulcanologia (INGV-OE)^56^. Remote sEnsing obServations of source Parameters and data IntegRation at EtnA: the RESPIRA database (Version 2) [Data set]. Istituto Nazionale di Geofisica e Vulcanologia (INGV). https://doi.org/10.13127/etna/respira-v2).

The RESPIRA repository contains the main ESPs, corresponding to 141 Etna eruptions, from 1986 to 2025, as listed in the first column of the dataset (one eruption per row). For each event, we specify the crater in the second column. The third and fourth columns report the onset and the duration of the paroxysmal phase. The fifth column lists the maximum CH while the sixth the maximum MER values. The TM is listed in the seventh column, in the height column there is the mode or mean values of TGSD while references are reported in the last column.

To help the reader identify the source of the data in each row of RESPIRA dataset, different colors are used to distinguish between literature and sensor/model derived data.

**Fig. 3:**
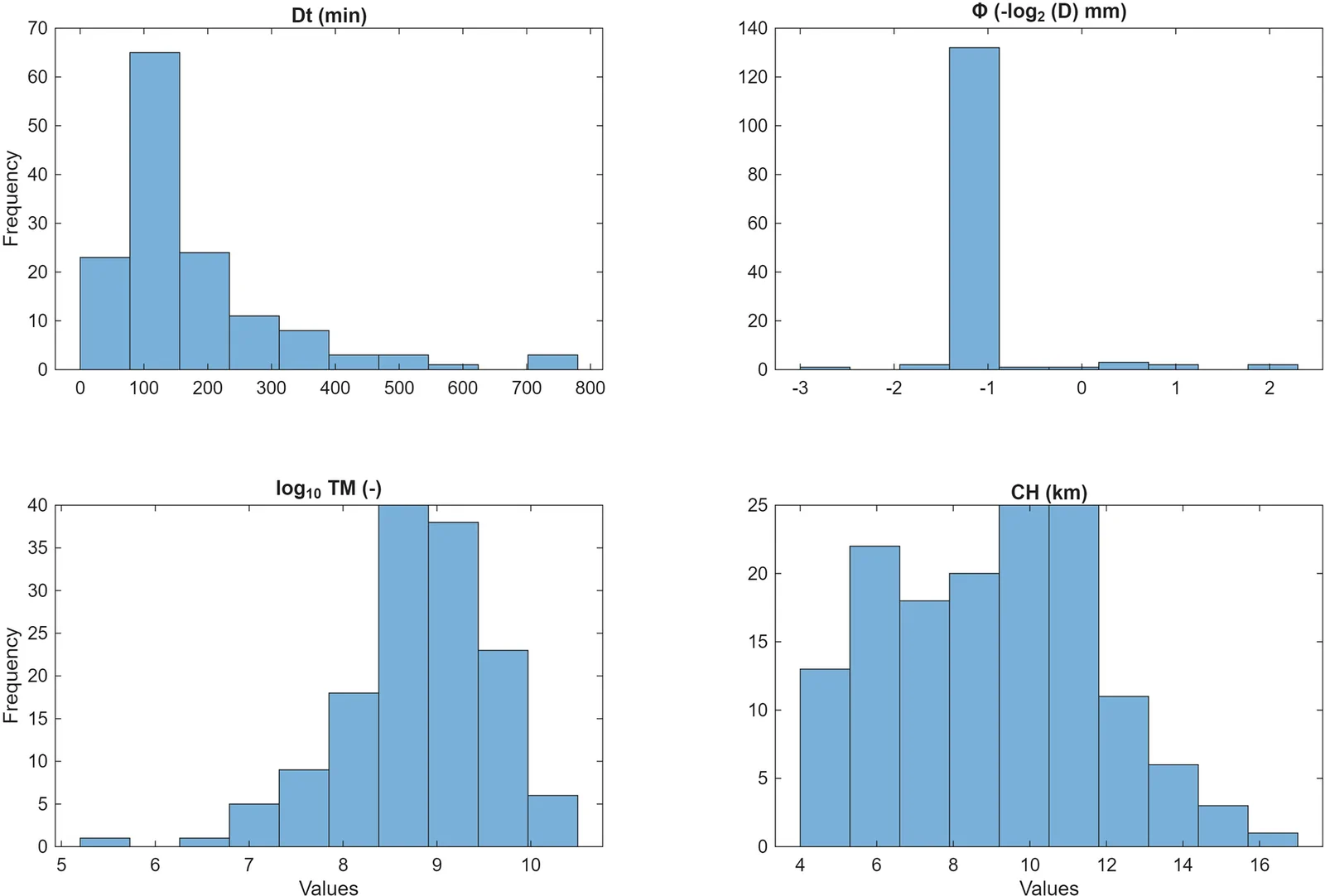
Frequency Distribution of four main ESPs listed in the RESPIRA database: Dt (min) and φ (−) are shown in the top- left and top-right panels, respectively, while log_10_(TM) and CH (km) are displayed in the bottom-left and bottom-right panels.

Figure 3 presents the histograms for the four ESPs: (1) Dt (min) which exhibits a logarithmic decreasing trend as the duration of the eruption increases; (2) φ showing a grain-size variability between −3 and 2.4 with a peak at −1, primarily influenced by the model employed^44^; (3) log_10_(TM) with values ranging between 4 10^6^ and 8 10^9^ kg; and 4) CH (km), which shows a distribution between 4 and 13 km a.s.l.

## Technical Validation

In order to validate the RESPIRA dataset, we consider the ESPs of Etna paroxysms of 10 April 2011 and 23 November 2013 because they were two events well observed with different sensors (cameras and radars) and observed in the field. The key ESPs, relating to Etna activity on 10 April 2011 and 23 November 2013 listed in Tables 2, 3, respectively, are: Dt (min), max MER (10^6^ kg/s), TM (10^8/9^ kg), CH (km) and TGSD (φ). The first row, highlighted in yellow, lists the data as shown in the RESPIRA dataset. For each of the five parameters, the various estimates are shown in order to assess their degree of similarity and order of magnitude.

**Table 2:** ESPs derived from different sensors and in the field for the Etna paroxysm of 10 November 2011, including the percentage difference with respect to values of the RESPIRA database.

Etna 10-04-2011	Dt (min)	max MER (10^6^ kg/s)	TM (10^8^ kg)	CH (km)	TGSD (φ)
RESPIRA	250	0.12	6.4	9	0.82^b^
Radar VDR	250^a^	0.08 33%	2.8^b^ 56%	—	—
Radar DPC	380^a^ 52%	0.08^a^ 33%	6.4^b^	9^a^	—
VIS/TIR Cameras	240^a^ 4%	0.12a —	1.2^b^ 81%	9^b^	—
Deposit	310^a^ 24%	—	0.25^b^ 96%	—	0.82^b^

a^14^,b^59^.

**Table 3:** Same as Table 2 but for the Etna paroxysm of 23 November 2013.

Etna 23-11-2013	Dt (min)	max MER (10^6^ kg/s)	TM (10^9^ kg)	CH (km)	TGSD (φ)
RESPIRA	70	2.3	3.9	12.8	−3^f^
Radar VDR	50^a^ 29%	—	3.7^a^ 5%	—	—
Radar DPC	60^b^ 14%	2.3^b^	3.9^b^	12.8^b^	2^f^ 18%
VIS/TIR Cameras	60^b^ 14%	1.8^b^ 22%	4.7^b^ 20%	>9^c^	—
Deposit	50^d^ 29%	1.9^e^ 17	1.3^d^ 67%	11^d^ 12%	−3^f^76%

a^14^; b^54^; c^24^; d^60^; e^43^, f^45^.

For the 10 April 2011 event (Table 2), all methods exhibit durations on the order of hours, ranging from 240 to 380 minutes. Regarding the maximum MER estimates, values spanned between 10^4 ^kg/s and 10⁶ kg/s. Notably, radar-derived estimates were approximately 33% lower than those from VIS/TIR cameras. The TM values have the same order of magnitude (10^8^ kg), with the greatest estimate obtained with radar VDR, while the TM obtained by the analysis of the deposit is one order of magnitude lower. Both DPC radar and VIS camera detect a CH of 9 km a.s.l. and the TGSD calculated from the deposit was 0.82 φ.

As illustrated in Table 3, all methods yielded consistent durations for the 23 November 2013 event, ranging from 50 to 70 minutes. With regard to the maximum MER estimates, the order of magnitude is 10⁶ kg/s, with the highest values derived from the radar DPC but within 22% respect to the VIS/TIR cameras. The TM values have the same order of magnitude (10⁹ kg), with the greatest difference between radar DPC and deposit (67%). CH ranges between 9 and 12.8 km a.s.l. (<12%). Regarding the TGSD, the values show very high uncertainty ranging between −3 and 2 φ, confirming that different sensitivity of sensors and methodologies applied play a crucial role and that future investigations are still needed to constrain this variability. Furthermore, for approximately 90% of the paroxysmal events, we found TGSD equal to −1 φ estimated using modelling approach^44^. This suggests that the model may not fully account for the natural variability inherent in the TGSD of Etna’s paroxysmal activity.

## Data Availability

The RESPIRA is freely available in the INGV repository under the Creative Commons Attribution 4.0 International (CC BY 4.0) license (https://doi.org/10.13127/etna/respira-v2).

## Code availability

No specific code was developed for this study.
